# *Salvia castanea* Hairy Roots are More Tolerant to Phosphate Deficiency than *Salvia miltiorrhiza* Hairy Roots Based on the Secondary Metabolism and Antioxidant Defenses

**DOI:** 10.3390/molecules23051132

**Published:** 2018-05-10

**Authors:** Lin Liu, Dongfeng Yang, Bingcong Xing, Haihua Zhang, Zongsuo Liang

**Affiliations:** 1 College of Life Science, Northwest A&F University, Yangling 712100, China; liulinlin0101@sina.com; 2 School of Life Science, Zhejiang Sci-Tech University, Hangzhou 310018, China; yangdongfeng@zstu.edu.cn (D.Y.); hhzhang@zstu.edu.cn (H.Z.); 3 Institute of Soil and Water Conservation, CAS & MWR, Yangling 712100, China; xingbingcong@163.com

**Keywords:** antioxidants, bioactive components, phosphate deficiency, *Salvia miltiorrhiza*, secondary metabolites, SPX (Syg1-Pho81-Xpr1)

## Abstract

*Salvia miltiorrhiza* is a well-known traditional Chinese herb which is used to treat heart disease. *Salvia castanea* is a substitute product for *S. miltiorrhiza* in the medicinal field. Previous study has shown that phosphate (Pi) deficiency could promote the accumulation of secondary metabolism in herbs, and it has also developed a strategy for saving Pi resources and increasing the yield of active substances in herbs. In the present study, the hairy roots of *S. miltiorrhiza* and *S. castanea* were used to identify the Pi deficiency response mechanisms of these two *Salvia* species. The results showed that Pi deficiency increased the accumulation of specifically secondary metabolites, such as phenolic acids and tanshinones, which were caused by promoting the expression levels of key enzyme genes. In addition, Pi deficiency promoted the antioxidant activity in these two *Salvia* species. The data demonstrated that Pi deficiency increased the quality of the medicinal material in the plant. The hairy roots of *S. castanea* were more adaptive to Pi deficiency than those of *S. miltiorrhiza* in terms of biomass, secondary metabolism, and antioxidant activity. The results of this study provide insights into breeding herbs that are better adapted to Pi deficiency, which could increase the yield of active ingredients in herbs and save Pi resources.

## 1. Introduction

Phosphorus is an essential nutrient for the growth and development of plants. However, only 20–50% of total soil phosphorus is available as inorganic phosphate (Pi) and the rest is converted to organic compounds, which are insoluble and unavailable to plants, leading to Pi starvation around the world [1,2,3,4]. Plants have evolved a series of adaptive responses to cope with Pi limitations: the symbiotic association with arbuscular mycorrhiza to enhance the Pi utilization rate; the increase in lateral roots; and root hairs to augment the Pi uptake area for acquiring more Pi from the environment [5,6]. This adaptive strategy is beneficial when breeding herbs whose roots are used as medicine.

Metabolic changes are also critical for plants’ adaptation to Pi deficiency [7]. The primary metabolism remodeling is a well-studied metabolic adaptation to low-Pi stress in plants [8]. Genes and enzymes involved in primary sugar and lipid metabolism bypasses are strongly induced during prevailing Pi deficiency, and these metabolic adaptations serve to improve Pi acquisition from the soil and promote Pi use efficiency [9,10]. Secondary metabolism—which evolved from primary metabolism and plays a role in the interactions between plants and their environment—is also induced by a low-Pi condition [7]. The accumulation of anthocyanin in modern plants and active medicinal ingredients in herbs are promoted by Pi deficiency [11,12,13]. This adaptive strategy enables the breeding of Pi high-efficiency herbs, whose secondary metabolism has a huge medicinal value.

The dried roots of *Salvia miltiorrhiza* Bunge have been used for the treatment of myocardial infarction, heart disease, and cerebrovascular diseases [14,15]. The major active compounds in *S. miltiorrhiza* are phenolic acids (such as caffeic (CA), salvianolic B (LAB), and rosmarinic acids (RA)) and tanshinones (such as tanshinone I (TI), tanshinone IIA (IIA), dihydrotanshinone I (DT), and cryptotanshinone (CT)) [16]. Improving the production of these compounds and finding substitute products are critical due to the low amounts normally found in *S. miltiorrhiza* [16]. *Salvia castanea* Diels f. *tomentosa* Stib is a sibling species of *S. miltiorrhiza*. *S. castanea* has the same bioactive tanshinones and phenolic acids as *S. miltiorrhiza* and served as a substitute product of *S. miltiorrhiza* for the therapy of diseases. *S. castanea* can grow at an elevation of 2500–3750 m in a harsh environment, which indicates that *S. castanea* has more sophisticated mechanisms to adapt to various stresses [17].

In *Salvia*, phenolic acids are mainly synthesized by the phenylpropanoid and tyrosine-derived pathways. Phenylalanine ammonia lyase (PAL) and tyrosine aminotransferase (TAT) are the rate-limiting enzymes in these pathways, respectively. Rosmarinic acid synthase (RAS) and cytochrome P450-dependent monooxygenase (CYP98AH14) are the key enzymes in the downstream of phenolic acid biosynthesis [18]. Tanshinones are mainly synthesized by the mevalonate (MVA) and 2-C-methyl-d-erythritol-4-phosphate (MEP) pathways. DSX1 (1-deoxy-d-xylulose) and 3-hydroxy-3-methylglutary CoA reductase (HMGR) are the rate-limiting enzymes in these pathways, respectively. Kaurene synthase-like (KSL) and CYP76AH3 are the key enzymes in the downstream of tanshinones biosynthesis [19]. The expressions levels of the genes encoding these key enzymes may suggest changes in enzyme/protein abundance and activities, and then influence the rate of synthesis of phenolic acids and tanshinones to some extent [19,20]. A previous study showed that Pi deficiency could promote the accumulation of phenolic acids in *S. miltiorrhiza* [21]. However, the effect of Pi deficiency on tanshinones biosynthesis, the effect of Pi deficiency on the expression levels of key enzyme genes, and the differences between the adaptive mechanisms of *S. miltiorrhiza* and *S. castanea* remain to be determined.

Genes involved in secondary metabolism fall into the late transcriptional phosphate starvation-inducible (*PSI*) genes, and the genes encoding the SPX (Syg1-Pho81-Xpr1) domain-containing proteins are the early *PSI* genes [7]. *SPX* senses low-Pi stress and activates Pi deficiency responses by regulating some late transcriptional *PSI* genes [5,22,23]. The expression level of *SPX* reflects the response to Pi deficiency to a degree. To investigate the differences between the adaptive mechanisms of *S. miltiorrhiza* and *S. castanea* in response to Pi deficiency, the Pi concentration and expression level of *SPX* were measured.

Environmental changes contribute to the differences in antioxidant activity of medicinal plants, the rich antioxidant activity can significantly affect their effectiveness as medicines and functional food supplements [24,25]. Therefore, antioxidant activity should be regarded as an additional health-promoting property of phytonutrients [26]. Both phenolic acids and tanshinones provide important sources of antioxidants [26,27]. Few reports are available on the relationship between Pi deficiency and antioxidant activity in herbs.

Hairy root cultures provide a rapid and simplified system, and represent a traditional approach for the development and study of the secondary metabolism in medicinal plants [28]. In the present study, *S. miltiorrhiza* and *S. castanea* hairy roots were used as material. The present study aims to clarify the effect of Pi deficiency on the production of secondary metabolism and antioxidant activity in *Salvia*, and identify differences in the Pi-deficient response mechanisms of *S. miltiorrhiza* hairy roots and *S. castanea* hairy roots. 

## 2. Results and Discussion

### 2.1. Biomass of Hairy Roots under Different Pi Concentrations

**Figure 1:**
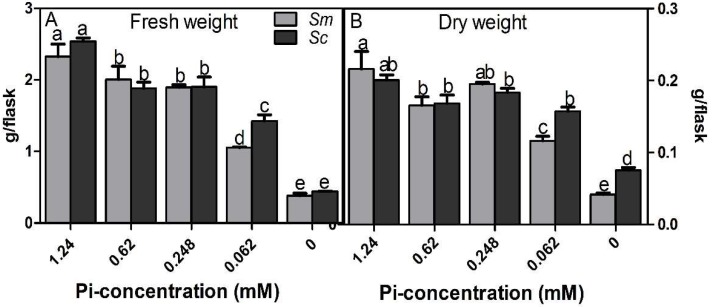
Biomasses of hairy roots in different inorganic phosphate (Pi) concentration conditions. (**A**) The fresh weight. (**B**) The dry weight. *Sm*, *S. miltiorrhiza*; *Sc*, *S. castanea*. Different letters indicate significant differences by applying Duncan’s multiple range test.

As shown in Figure 1, the biomass of *S. miltiorrhiza* hairy roots and *S. castanea* hairy roots were affected by the different Pi dosages (*p* < 0.05). The fresh weight decreased significantly as the Pi concentration decreased, especially in the 0 mM Pi condition (Figure 1A). The dry weight of *S. castanea* hairy roots was minimally influenced by Pi dosages, except for the 0 mM condition. However, the dry weight of *S. miltiorrhiza* hairy roots was decreased significantly in the 0.062 mM and 0 mM Pi conditions (Figure 1B). 

Low Pi availability stimulates root hair elongation in many plants [6,29], which could present an opportunity for plants whose roots are used medicinally, such as the well-known herbs *S. miltiorrhiza* and *S. castanea* [30,31]. In this study, the dry weight of the hairy roots did not significantly decrease following the treatment of *S. miltiorrhiza* with 0.248–1.24 mM Pi or *S. castanea* with 0.062–1.24 mM Pi (Figure 1). The lack of change in dry weight could arise because Pi deficiency may diminish some physiological activity, such as the inhibition of primary root growth [32]. However, plants can enhance their lateral root and root hair production to increase Pi uptake as an adaptive response to the deficiency [7,33]. As a result, these two factors could have balanced each other out and prevented difference in dry weight at different Pi levels. The biomass of *S. castanea* hairy roots showed less change than that of *S. miltiorrhiza* hairy roots, especially for the dry weight.

### 2.2. Effects of Pi Deficiency on the Content and Yields of Specialized Metabolites in Hairy Roots

**Figure 2:**
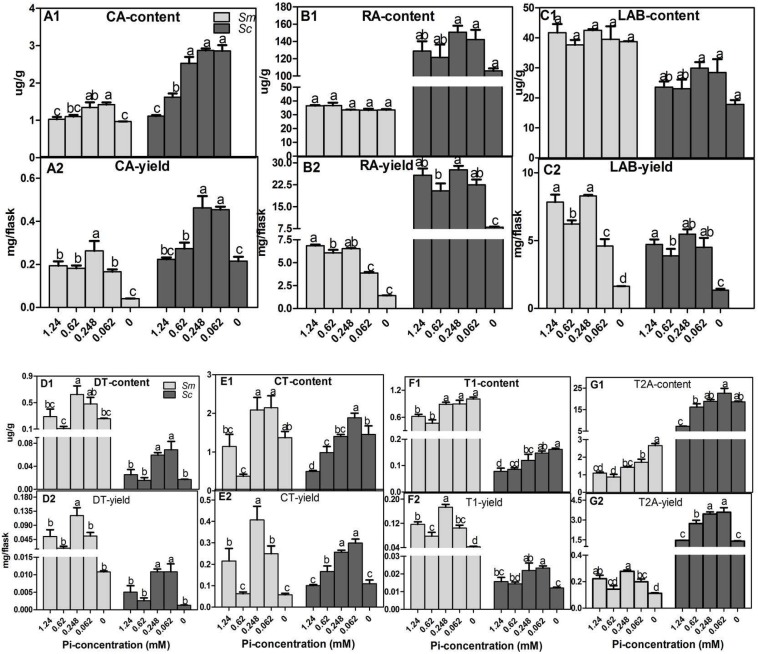
Effects of Pi deficiency on the content and yield of secondary metabolites in hairy roots. (**A1**,**A2**) Content and yield of CA. (**B1**,**B2**) Content and yield of RA. (**C1**,**C2**) Content and yield of LAB. (**D1**,**D2**) Content and yield of DT. (**E1**,**E2**) Content and yield of CT. (**F1**,**F2**) Content and yield of TI. (**G1**,**G2**) Content and yield of T2A. Different letters indicate significant differences by applying Duncan’s multiple range test. *Sm*, *S. miltiorrhiza*; *Sc*, *S. castanea*; CA, caffeic acid; LAB, salvianolic acids B; RSA, rosmarinic acids; TI, tanshinone I; IIA, tanshinone IIA; DT, dihydrotanshinone I; CT, cryptotanshinone.

The secondary metabolites, including three phenolic acids (CA, RA, LAB) and four tanshinones (DT, CT, TI, T2A), were measured. In the 1.24 mM Pi treatment (control group), *S. miltiorrhiza* accumulated higher levels of LAB (Figure 2C), DT (Figure 2D), and TI (Figure 2F) but had lower levels of CA (Figure 2A), RA (Figure 2B), and T2A (Figure 2G) than those of *S. castanea*, which agrees with our previous work [30,34].

Pi deficiency had minimal effects on the accumulation of RA and LAB in both *Salvia* species (Figure 2B,C). However, the accumulation of CA (Figure 2A) and four tanshinones (Figure 2D–G) were promoted under Pi deficiency. The contents of CA (Figure 2A1), DT (Figure 2D1), CT (Figure 2E1), TI (Figure 2F1), and T2A (Figure 2G1) in *S. miltiorrhiza* hairy roots in the 0.062 mM Pi treatment condition were 1.33, 1.66, 1.87, 1.45, and 3.12 times greater than the contents at the 1.24 mM Pi treatment condition. Furthermore, there were no significant differences across the yields (Figure 2D2–G2). The contents of CA, DT, CT, TI, and T2A in *S. castanea* hairy roots in the 0.062 mM Pi treatment condition were 2.58, 2.76, 3.76, 1.86, and 3.12 times greater than the 1.24 mM condition. The yields of CA, DT, CT, TI, and T2A were 2.04, 2.2, 2.98, 1.48, and 2.45 times greater than the 1.24 mM Pi treatment condition. The data suggested that Pi deficiency promoted the accumulation of specialized metabolites in *Salvia*, and the promotion amount in *S. castanea* hairy roots was larger than that in *S. miltiorrhiza* hairy roots.

The quality of traditional medicine is determined by the active substances produced by the medicinal plants. The accumulation of secondary metabolites is an adaptive strategy for plants in response to Pi deficiency. The accumulations of anthocyanin in *Arabidopsis* [35] and rice [36], the accumulations of ginsenosides in *Panax ginseng* [37], and the accumulations of saikosaponin in *Bupleurum chinense DC* [13] are promoted by Pi deficiency. Many secondary metabolites are likely to play defensive roles in protecting themselves from biotic and abiotic stress. Pi deficiency promoted the accumulation of secondary metabolites, which increased the adaptive capacity of *Salvia* to Pi deficiency. Indeed, the promotion degree of secondary metabolism in plants reflects the adaptive capacity to Pi deficiency. The promotion amount in *S. castanea* hairy roots was larger than that in *S. miltiorrhiza* hairy roots, which demonstrated that *S. castanea* hairy roots could remodel secondary metabolism more effectively to adapt to Pi deficiency than *S. miltiorrhiza* hairy roots.

Taking into account the biomass of hairy roots and the amounts of the seven secondary metabolites across the different Pi treatment concentrations, we found that the 0.062 mM Pi condition had small effect on the biomass, while having a beneficial effect on the yield of secondary metabolism. Therefore, 0.062 mM Pi treatment condition was selected for the further study.

### 2.3. Effects of Pi Deficiency on the Quantification of the Transcript Levels of Key Enzyme Genes in Hairy Roots

To determine whether the enhanced accumulation of secondary metabolites and the differences in these two *Salvia* species were due to the transcript level of key enzyme genes in synthetic pathways or not, the expression level of key enzyme genes involved in phenolic acid and tanshinone biosynthesis were measured.

**Figure 3:**
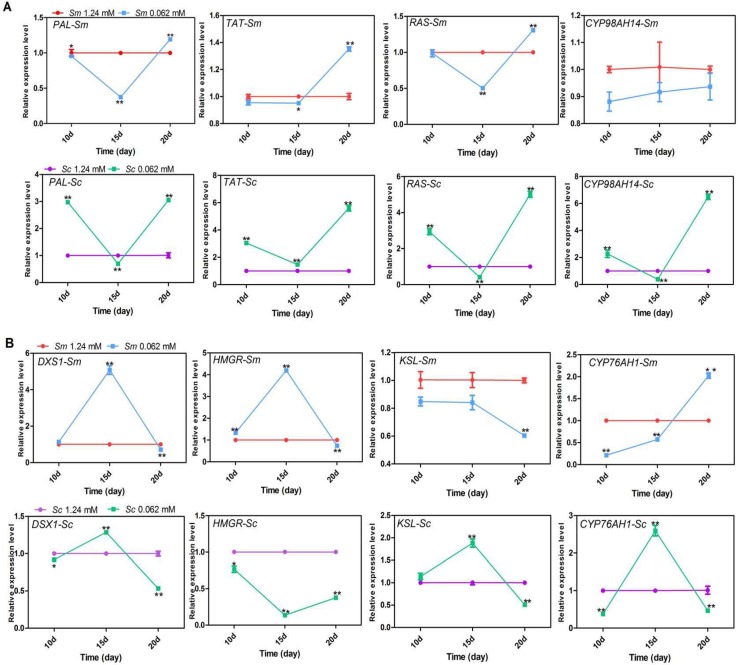
Quantification of the transcript levels of key enzyme genes in hairy roots under control and Pi-deficient conditions at different times (10th, 15th, 20th day). Quantification of the transcript levels of key enzyme genes were calculated in relation to *β-actin* reference gene transcript levels. (**A**) Genes involved in phenolic acid biosynthesis. (**B**) Genes involved in tanshinone biosynthesis. *Sm*, *S. miltiorrhiza*; *Sc*, *S. castanea*; *PAL*, phenylalanine ammonia lyase; *TAT*, tyrosine aminotransferase; *RAS*, rosmarinic acid synthase; *CYP98A14*, cytochrome P450-dependent monooxygenase; *DXS1*, 1-deoxy-d-xylulose 5-phosphate 1; *HMGR*, 3-hydroxy-3-methylglutary CoA reductase; *KSL*, kaurene synthase-like; *CYP76AH1*, cytochrome P450-dependent monooxygenase. The asterisk “*”represents 0.01 < *p* < 0.05; asterisks “**”represents *p* < 0.01 compared to the control phosphate value and obtained via Student’s *t*-test by SPSS.

The results showed that in Pi-deficient conditions, except for *CYP98AH14* in *S. miltiorrhiza*, the transcript level of genes in the phenolic acid pathway reached the highest on the 20th day in these two *Salvia* species (Figure 3A). In contrast, except for *CYP76AH1*, transcript levels of genes in the tanshinone pathways reached the highest on the 15th day in these two *Salvia* species under Pi deficiency (Figure 3B). These data indicated that the accumulation of phenolic acids and tanshinone may have occurred at different stages.

The expression levels of genes in the *S. castanea* phenolic acid biosynthetic pathways were more responsive to Pi deficiency than those in *S. miltiorrhiza* (Figure 3A). For example, after 20 days of Pi deficiency, the expression levels of *PAL*, *TAT*, *RAS*, and *CYP98AH14* had negligible effects in *S. miltiorrhiza*, however, they were promoted by more than 3.0, 5.6, 5.02, and 6.48 times than that of control levels in *S. castanea*.

*CYP98AH14* and *KSL* showed different change trends in these two *Salvia* species. In the Pi deficiency condition, expression levels of *CYP98AH14* and *KSL* were increased in *S. castanea* most of the time, while they were inhibited in *S. miltiorrhiza* throughout the experiment. The different expression patterns of these two genes may contribute to the differences between the adaptive mechanisms of *S. miltiorrhiza* and *S. castanea* in response to Pi deficiency.

The accumulations of phenolic acids and tanshinones are the results of the function of enzymes in biosynthetic pathways [38]. In the present study, Pi deficiency upregulated the expressions of key enzyme genes in the biosynthetic pathways of tanshinones and phenolic acids at specific times, which likely resulted in their accumulation. This result is consistent with previous reports [9,10,21]. Genes in *S. castanea* were more responsive to the low-Pi stress than those in *S. miltiorrhiza*, which is consistent with the greater degree of content change of secondary metabolism in *S. castanea* compared to *S. miltiorrhiza.* The genes involved in secondary metabolism were late transcriptional *PSI* genes, and were regulated by early *PSI* genes [39,40]. 

### 2.4. Expression Response of the SPX Genes to Pi Deficiency

**Figure 4:**
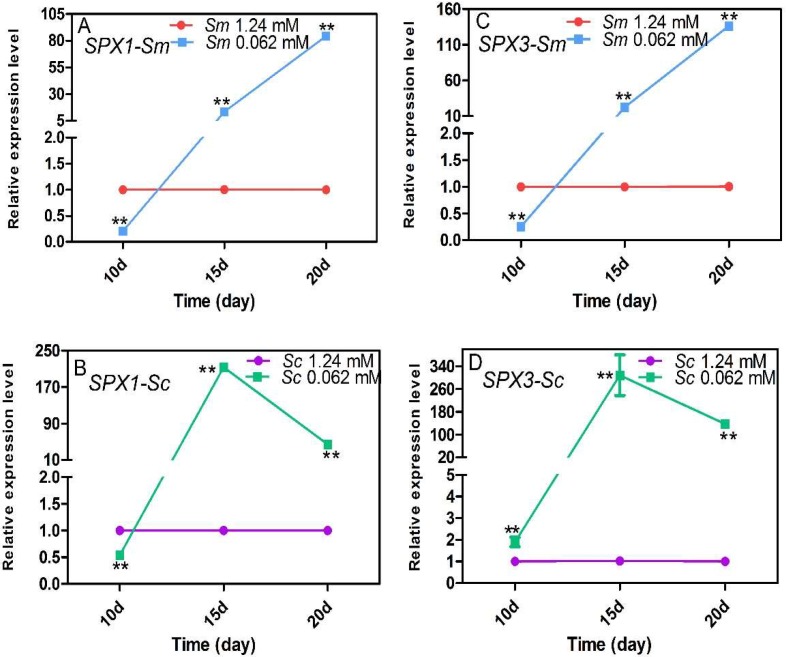
Expression of *SPX1* and *SPX3* in *S. miltiorrhiza* (**A**,**C**) and *S. castanea* (**B**,**D**) under control and Pi-deficient conditions. Quantification of the transcript levels of *SPX* genes were calculated in relation to *β-actin* reference gene transcript levels. *Sm*, *S. miltiorrhiza*; *Sc*, *S. castanea*; *SPX*, *Syg1-Pho81-Xpr1*. The double-asterisk “**”represents *p* < 0.01 compared to the control Pi value and obtained via Student’s *t*-test by SPSS.

We measured the expression levels of *SPX* genes to reveal the different responses to Pi deficiency in *S. miltiorrhiza* and *S. castanea*. The full sequences of *SPX1* (Gene ID: MH155196) and *SPX3* (Gene ID: MH155198) were obtained from the transcriptome and were detected by gene clone. In the Pi-deficient condition, *SPX1* in *S. miltiorrhiza* (Figure 4A) and *S. castanea* (Figure 4B), as well as *SPX3* in *S. miltiorrhiza* (Figure 4C), were inhibited on the 10th day but were promoted significantly on the 15th day 20th day. *SPX3* in *S. castanea* (Figure 4D) showed sustained induction in the whole process. The highest expression levels of *SPX1* and *SPX3* in *S. castanea* hairy roots were measured at 15 days and were 213 and 308.5 times greater than control levels, respectively. The highest expression levels of *SPX1* and *SPX3* in *S. miltiorrhiza* were measured on the 20th day and were 84.1 and 136 times greater than control levels, respectively. That is to say, *SPX1* and *SPX3 in S. castanea* had a more timely and sensitive response to Pi deficiency than those in *S. miltiorrhiza.*

SPXs are early response PSI genes that connect the Pi deficiency signal to the Pi deficiency response. SPXs promote the activity of phosphate transporters factor (PHT) to increase Pi transport [12,41,42], regulate secondary metabolism genes, among other activities [23,43,44,45]. The expression levels of SPXs reflected the degree of response to Pi deficiency to some extent. MEGA 6 software was used to perform multiple sequence alignment on *Salvia* SPX1 and SPX3 protein sequences, AtSPXs protein sequences [46,47], OsSPXs protein sequences [22,48], and GmSPXs protein sequences [49]. The result showed that SmSPX1 and AtSPX1 were clustered together and SmSPX3 and AtSPX3 were clustered together, respectively (Supplementary material Figure S1). AtSPX1 and AtSPX3 are strongly induced by Pi deficiency and play a positive role in plant adaptation by regulating the expression of the Pi-responsive genes in response to Pi starvation [45,46]. SmSPX1 and SmSPX3 were promoted to levels hundreds of times greater than control levels. These data demonstrated that *SPX1* and *SPX3* of *Salvia* might play a positive role in plant adaptation to Pi starvation. 

### 2.5. Pi Accumulation in Hairy Roots at Control and Pi Deficiency Conditions

**Figure 5:**
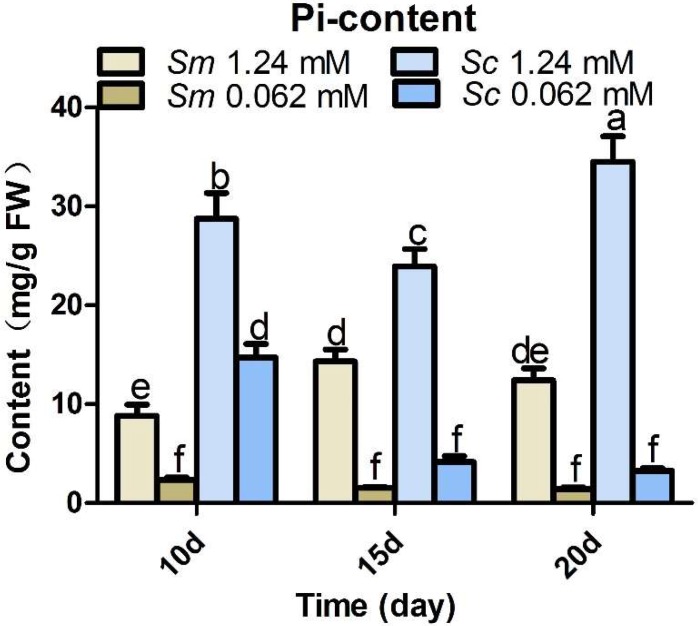
Pi concentration in hairy roots under control and Pi-deficient conditions. *Sm*, *S. miltiorrhiza*; *Sc*, *S. castanea*; different letters indicate significant differences by applying Duncan’s multiple range test.

To investigate Pi absorption and utilization efficiency in the two *Salvia* species, we measured the effective Pi content in *S. miltiorrhiza* hairy roots and *S. castanea* hairy roots under control and Pi-deficient conditions (Figure 5). Pi accumulation decreased markedly under Pi deficiency in both *Salvia* hairy roots. In *S. miltiorrhiza* hairy roots, the Pi concentration in the control group was 3.74, 8.8, and 8.9 times greater than in the various Pi-deficient groups on the 10th, 15th, and 20th day. In *S. castanea* hairy roots, the Pi concentration in the control group was 1.95, 5.8, and 10.6 times greater than the Pi-deficient groups on the 10th, 15th, and 20th day. The difference between the control group and the Pi-deficient group increased as the experiment progressed, possibly because the control group was able to uptake more Pi from the medium to sustain growth, while there was not enough Pi for the hairy roots of plants in the Pi-deficient groups. 

In general, the *S. castanea* experimental and control groups contained more effective Pi than the *S. miltiorrhiza* groups, which indicated that *S. castanea* can utilize Pi more efficiently than *S. miltiorrhiza*. *S. castanea* needed more Pi to sustain normal growth and had a more sensitive response to Pi deficiency, which could make it more accessible to Pi uptake. It is necessary to develop a better understanding of the molecular events involved in controlling Pi homeostasis in plants.

### 2.6. Antioxidant Enzyme Responses to Pi Deficiency

**Figure 6:**
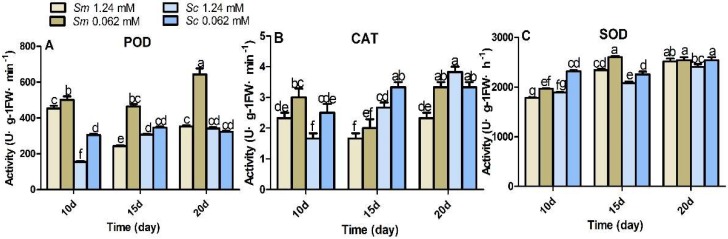
Antioxidant enzyme activities in hairy roots under control and Pi-deficient conditions. *Sm*, *S. miltiorrhiza*; *Sc*, *S. castanea*; different letters indicate significant differences by applying Duncan’s multiple range test.

Antioxidant enzyme activities were increased in samples treated with Pi deficiency (Figure 6). After 10 days in the *S. miltiorrhiza* samples, the activities of POD, CAT, and SOD were increased by 10%, 29%, and 10%, respectively. After 10 days, in the *S. castanea* samples, the activities of POD, CAT, and SOD were increased by 20%, 50%, and 98%, respectively. However, as the experiment progressed, the induction degree decreased gradually until it was extinct. This happened after 20 days for SOD, POD, and CAT in *S. castanea* and for SOD in *S. miltiorrhiza*.

Stress conditions could promote the production of reactive oxygen species (ROS), and the induction of antioxidant systems protects the plants from oxidative damage [50,51]. In the present study, the activities of the antioxidant enzymes were induced to adapt to Pi deficiency. The degree of induction decreased gradually overtime until extinction (Figure 6), which was an adaptive process. *S. castanea* was more reactive than *S. miltiorrhiza* in both the control and Pi-deficient groups. 

### 2.7. Effects of Pi Deficiency on the Antioxidant Activity of Salvia Hairy Roots

**Figure 7:**
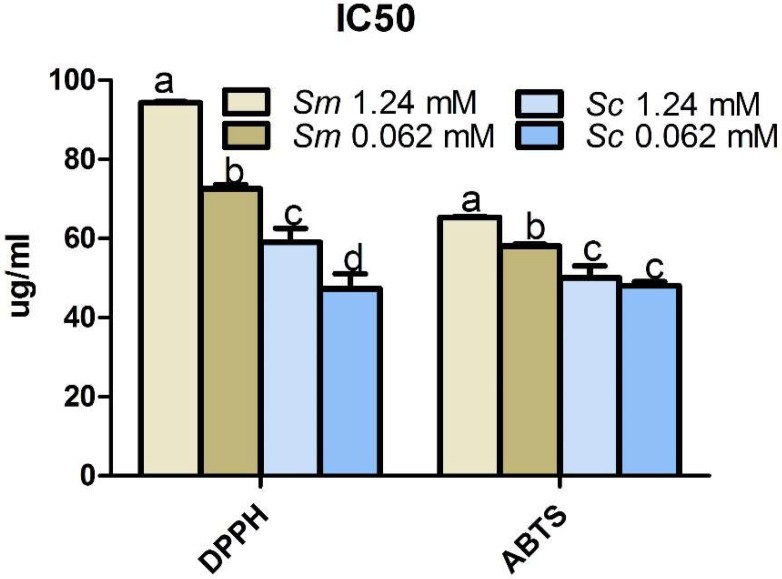
DPPH radical scavenging and ABTS radical scavenging of hairy roots under control and Pi-deficient conditions. *Sm*, *S. miltiorrhiza*; *Sc*, *S. castanea*; different letters indicate significant differences by applying Duncan’s multiple range test.

To investigate the influence of Pi deficiency on the antioxidant activity of *Salvia* hairy roots, DPPH radical scavenging activity and ABTS^+^ radical cation scavenging activity were measured (Figure 7). A lower DPPH or ABTS^+^ IC_50_ represented stronger antioxidant capacity. The radical scavenging ability of *S. miltiorrhiza* and *S. castanea* were induced by Pi deficiency. *S. castanea* showed stronger antioxidant ability than *S. miltiorrhiza* in both the control and Pi-deficient groups. 

Secondary metabolism exerts antioxidation effects. Antioxidation characteristics reflect the quality of the medicinal material to some degree. Many reports strongly suggest that environmental conditions influence the antioxidant properties of plants [25,26,50]. In the present study, the DPPH scavenging capacity assay and ABTS^+^ radical scavenging assay promoted by Pi deficiency might not fully reflect the antioxidant properties of *Salvia* (Figure 7). Thus, further studies in cells and living organism are required. *S. castanea* had greater antioxidant activity than *S. miltiorrhiza* in both control and Pi-deficient groups. These data are consistent with the accumulation of secondary metabolites and provide further evidence that *S. castanea* was more amenable to Pi deficiency compared to *S. miltiorrhiza*.

**Figure 8:**
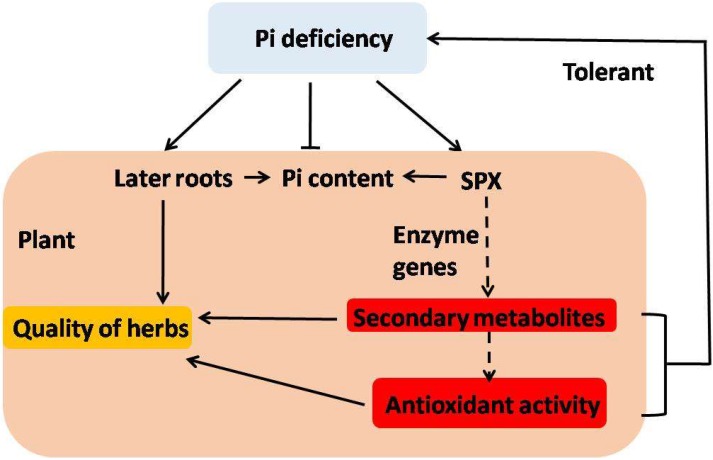
The model of interaction of plants with Pi deficiency based on secondary metabolism and antioxidant defenses. Uncertain steps are represented by dotted lines.

The chemical compositions and antioxidant activity in medicinal plants may lead to significant differences in effectiveness as medicines, functional foods, and nutritional supplements. Pi deficiency should be considered as a strategy to improve the quality of traditional Chinese herbal medicine (Figure 8). *S. castanea* hairy roots was more adaptive to Pi deficiency than *S. miltiorrhiza* hairy roots, likely because of the *S. castanea* growing environment, which is harsh compared to that of *S. miltiorrhiza* [30]. This environmental difference may make *S. castanea* more capable of adapting to stressors. Our findings suggest the possibility of optimizing growing conditions for Pi high-efficiency in plants, with desired antioxidant properties.

## 3. Materials and Methods

### 3.1. Plant Material and Pi Deficiency Treatment

Two-year-old seeds of *S. miltiorrhiza* were obtained from the experimental field at Northwest A&F University, and 2-year-old seeds of *S. castanea* were collected from the Science and Technology Bureau of Nyingchi Prefecture, Tibet, People’s Republic of China. These seeds were sterilized and cultured on Murashige and Skoog solid medium to obtain sterile plantlets. Sterile plantlets were grown for 3 months. The *Agrobacterium rhizogenes* strain ATCC 15834 was applied to infect *S. miltiorrhiza* and *S. castanea* aseptic leaves to obtain hairy roots, and the hairy roots were subcultured every 30 days [28]. Hairy roots (0.2 g, fresh weight) were cultured in 100 mL flasks that contained 50 mL of 6, 7-v medium on an orbital shaker. The shaker was set at 110 rpm at 25 °C in the dark. The control group (CK) of Pi from NaH_2_PO_4_ in the 6, 7-v medium was 1.24 mM. 

Five levels of Pi treatment (1.24 mM, 0.62 mM, 0.248 mM, 0.062 mM, and 0 mM) were used, corresponding to a control group, a slightly reduced level, a moderately reduced level, a seriously reduced level, and the complete absence of Pi, respectively.

For biomass and high-performance liquid chromatography (HPLC) measurement, the hairy roots grown at the five phosphate treatment levels were harvested after 28 days. The fresh weight of each sample was recorded, then, the samples were dried at 50 °C in an oven until constant dry weight for HPLC.

For Pi concentration measurement, hairy roots grown in 1.24 mM and in 0.062 mM Pi for 28 days were harvested to measure the concentration of Pi.

For quantitative real-time PCR (qRT-PCR) and antioxidant assays, the hairy roots were grown in the 1.24 mM and in 0.062 mM Pi levels, and hairy roots were collected at 10, 15, and 20 days, respectively. 

### 3.2. High-Performance Liquid Chromatography (HPLC)

The extraction methods for active components (CA, LAB, RA, TI, TIIA, DT, CT) and HPLC were carried out in accordance with the general method used previously in our laboratory [52]. Dry sample powder (20 mg) was dissolved in 2 mL of 70% methanol and soaked for 12 h. The mixture was sonicated for 45 min and centrifuged at 8000 rpm for 10 min. The supernatant was filtered through a 0.22 um microporous membrane (F) for HPLC analysis using a Waters e2695 Separations Module and 2998 PDA Detector. Chromatography separation was performed with a C18 column (Waters), the temperature of column was 30 °C. The detected wavelength was 288 nm for phenolic acids and 270 nm for tanshinones. The mobile phase was acetonitrile (C) and 0.026% phosphoric acid solution (D) with the flow rate of 1.00 mL/min. The elution gradient was set as follows: *t* = 0–10 min, 5–20% C; *t* = 10–15 min, 20–25% C; *t* = 15–20 min, 25% C; *t* = 20–25 min, 25–20% C; *t* = 25–28 min, 20–30% C; *t* = 28–40 min, 30% C; *t* = 40–45 min, 45% C; *t* = 45–58 min, 45–58% C; *t* = 58–67 min, 58–50% C; *t* = 67–70 min, 50–60% C; *t* = 70–80 min, 60–65% C; *t* = 80–85 min, 65–95 C; *t* = 85–95 min, 95 C; *t* = 95–96 min, 5% C. Compounds were identified by comparison with the standard substances, which were purchased from the National Institutes for Food and Drug Control (Beijing, China).

### 3.3. RNA Extraction, cDNA Synthesis, and Quantitative Real-Time PCR (qRT-PCR) Assays

Total RNA was isolated from the fresh hairy root tissues (100 mg) using the RNAprep pure Plant Kit (Tiangen, Beijing, China). RNA (1 µg) was used to synthesize cDNA using the PrimeScript™ RT reagent kit with gDNA Eraser (Perfect Real Time, Takara, Tokyo, Japan) according to manufacturer’s instructions. The expression levels of genes were detected by qRT-PCR using SYBR^®^ Premix Ex Taq™ II (Takara, Japan) using QuantStudio 6 Flex Real-Time PCR System (Thermo Fisher, Waltham, MA, USA). The constitutively expressed *β-actin* gene was used as an internal reference gene to estimate the relative gene expression. Primers used for qRT-PCR was designed though the website of https://www.genscript.com/tools/real-time-pcr-tagman-primer-design-tool and are listed in Supplementary Table S1. The qRT-PCR conditions were: 95 °C for 30 s, 1 cycle; followed by 95 °C for 5 s; and 58 °C for 30 s, 40 cycles. The data was analysed by the delta-delta Ct method [53].

### 3.4. Measurement of Effective Pi Concentration

Effective Pi concentrations in hairy roots were performed using the molybdenum blue method, as described by Nanamori et al. [8]. Hairy roots (0.5 g, fresh weigh) were powder using liquid nitrogen, and the powder was homogenized in 1 mL of 10% (*w*/*v*) perchloric acid (PCA), using an ice-cold mortar and pestle. The homogenate was then diluted 10 times with 5% (*w*/*v*) PCA and placed on ice for 30 min. It was then centrifuged at 10,000× *g* for 10 min at 4 °C. The supernatant was used to measure the concentration of Pi. Ammonium molybdate (0.4%, *w*/*v*) was melted in 0.5 M H_2_SO_4_ (solution A) and then was mixed with 10% ascorbic acid (solution B; A:B = 6:1). Two milliliters of this solution was added to 1 mL of the sample solution, and the resulting solution was incubated in a water bath at 40 °C for 20 min. After being cooled in ice, the absorbance was measured at 820 nm. Conversion of the Pi content referenced the standard curve.

### 3.5. Analysis of Enzyme Activities

Hairy roots (0.2 g, fresh weight) were ground into a mortar using liquid nitrogen, and 2 mL of phosphate buffer (50 mM, pH 7.8, 0.1 mM EDTA, 1% PVP) was added to form a homogenate with 8 mL of phosphate buffer. The homogenate was centrifuged twice at 4 °C for 15 min at 12,000 rpm. The supernatant was retained for the analysis of enzyme activities, the method followed described as previous [50,54].

**Superoxide dismutase (SOD):** The reaction mixture contained 1.5 mL of phosphate buffer, 0.3 mL of 130 mM methionine, 0.3 mL of 750 μM nitroblue tetrazolium (NBT), 0.3 mL of 100 μM EDTA, 0.5 mL of water, 0.1 mL of enzyme extract, and 0.3 mL of 20 µM riboflavin. The chromogenic reaction occurred for 15 min under 4000 lx. Absorbance was measured at 560 nm. One unit of SOD activity was defined as the enzyme amount that suppressed the NBT photochemical reduction in half.

**Peroxidase (POD):** POD activity was determined by monitoring the increase in absorbance at 470 nm of a solution of 50 mM phosphate buffer (pH 5.5) containing 1 mM guaiacol, 0.5 mM H_2_O_2_, and 0.1 mL of enzyme extract. One unit of POD activity was defined as the amount of enzyme that increased the absorbance of a material by 0.01 per min. 

**Catalase (CAT):** CAT activity was measured by using H_2_O_2_ (extinction coefficient 39.4 mM cm^−1^) for 1 min at 240 nm. A reaction mixture (3 mL) that contained 1 mL Tris-HCl buffer (pH 7.0), 1.5 mL water, 0.2 mL enzyme extract, and 0.2 mL H_2_O_2_ (100 mM) was used.

### 3.6. DPPH and ABTS^+^ Radical Cation Screening Assay

Sample powder (20 mg, dry weight) was extracted with 70% methanol (2 mL) under sonication for 60 min, then centrifuged at 9000× *g* for 5 min. The supernatant (10 mg/mL) was then diluted 2, 4, 5, 6, and 12 times by using 70% methanol to form the gradient tested simples. We used published protocols by Liu to measure DPPH and ABTS^+^ radical scavenging activity [26,55]. 

The test samples (2 mL) were mixed with 2 mL of a DPPH solution (200 mM); the final concentration of DPPH was 100 mM. The mixture was shaken and allowed to react in the dark for 30 min. The absorbance was measured at 517 nm. IC_50_ values were defined as the effective concentrations at which DPPH radicals were scavenged by 50% and were obtained by interpolation from linear regression analysis.

The ABTS^+^ sample was prepared by mixing an ABTS^+^ stock solution (7 mM in water) with 2.45 mM of potassium persulfate. The ABTS^+^ solution was diluted with a phosphate buffered saline (PBS) solution (pH 7.4) to an initial absorbance of 0.7 at 734 nm. Tested samples (0.1 mL) were added to 3.9 mL of ABTS^+^ solution (OD = 0.7), and the absorbance was measured at 734 nm. IC_50_ values were defined as the effective concentrations at which ABTS^+^ radicals were scavenged by 50% and were obtained by interpolation from linear regression analysis.

### 3.7. Data Analysis

All experiments were repeated in triplicate, results are presented as means standard deviation (SD), all data were analyzed by using Statistical Package for Social science (IBM SPSS Statistics 19). Analyses were performed using one-way ANOVAs followed by the Duncan’s multiple range tests or Student’s *t*-test (indicated in the figure legends). Significant and highly significant levels were considered at 0.01 < *p* < 0.05 and *p* < 0.01, respectively. The figures were produced using GraphPad Prism 5.

## 4. Conclusions

Pi deficiency increased the accumulation of secondary metabolites and antioxidant activities, which improved the quality of the herbs (Figure 8). *S. castanea* hairy roots contained more Pi and was more responsive to Pi deficiency than *S. miltiorrhiza* hairy roots. The hairy roots culture could not fully represent the whole plant. Pi deficiency in whole plants may lead to undesirable losses, such as in biomass production, thus, losses in the yield of secondary compounds and crops yield can occur. In addition, results in this study raise the possibility of optimizing growing conditions of plants for the aspect of Pi high-efficiency, which could increase the yield of active compounds and save Pi resources. The research demonstrates that Pi fertilizer applied to agriculture should be based on different demands of agriculture. 

## Supplementary Materials

The following are available online at http://www.mdpi.com/1420-3049/23/5/1132/s1.
