# Supplementary material for: Salvia castanea Hairy Roots are More Tolerant to Phosphate Deficiency than Salvia miltiorrhiza Hairy Roots Based on the Secondary Metabolism and Antioxidant Defenses
Source: Molecules. 2018 May 10;23(5):1132. doi: 10.3390/molecules23051132 (PMC6099837; doi:10.3390/molecules23051132)
Supplement: Supplementary file 1 [file molecules-23-01132-s001.pdf]

Supplementary profiles

Table S1. Primers used for RT-qPCR

Gene	Sense Primer (5′–3′)	Reverse Primer (5′–3′)
β-ACTIN	GGTGCCCTGAGGTCCTGTT	AGGAACCACCGATCCAGACA
PAL	GGCGGCGATTGAGAGCAGGA	ATCAGCAGATAGGAAGAGGAGCACC
TAT	TTCAACGGCTACGCTCCAACT	AAACGGACAATGCTATCTCAAT
RAS	CGCCCTAGTTGAGTTCTACCCTTACGC	TCGGATAGGTGGTGCTCGTTTGC
CYP98AH4	CTAAGGAGGTGCTGAAGGAG	GTGGAGTCGTTGTAGATGGA
DXS1	CGACCAGGTAGTGCACGACG	TCATCTGAAGGAGCCATCACCAC
HMGR	GCAACATCGTCTCCGCCGTCTACA	GATGGTGGCCAGCAGCCTGGAGTT
KSL	CATGTCGAACAAGGACGTA	AATCATCCAAGGTTAGTGCC
CYP76AH1	CAGGAGGTGAACGGCTATCT	GTTATGAACCAGAGTCGCAGTAG
SPX1	ATGAGGGTGTGATCAAGGGA	ACTAATCTGGAGTGGTGGCAAT
SPX3	GCCAGACCACCATAGAATCG	GCGGCGGCAGAGAGAA

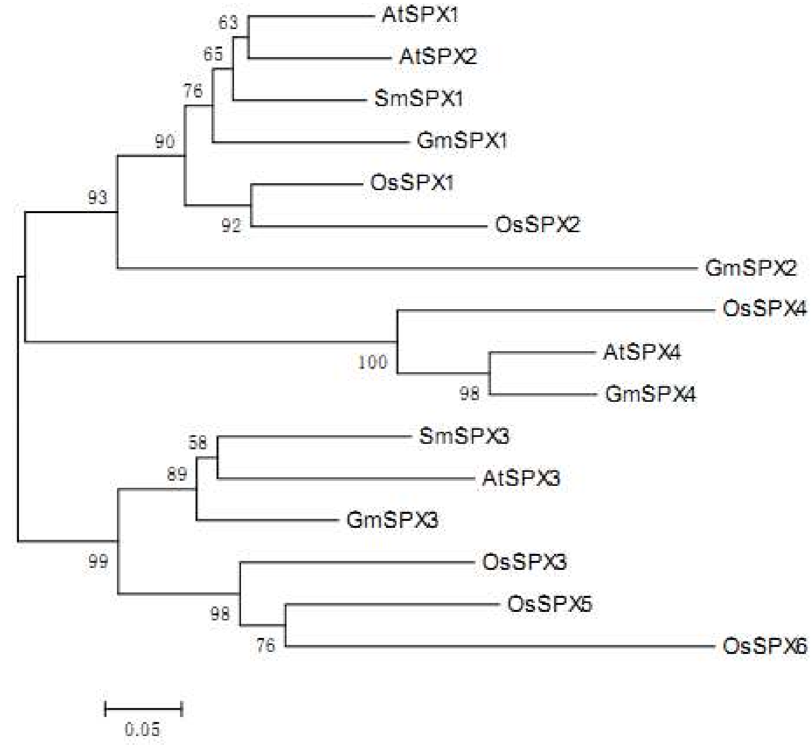

Figure S1. Multiple sequence alignment of SmSPX1/3 protein sequences with AtSPXs, OsSPXs and GmSPXs protein sequences.
